# Acute Poisoning Among Children Admitted to Alexandria Poison Center, Egypt: Patterns and Predisposing Factors

**DOI:** 10.7759/cureus.63720

**Published:** 2024-07-03

**Authors:** Esraa Abd El-Aziz, Mohamed M Tahoun, Moustafa A Arafa, Asmaa S El-Banna

**Affiliations:** 1 Epidemiology, High Institute of Public Health, Alexandria, EGY; 2 Forensic Medicine and Toxicology, Faculty of Medicine, Alexandria University, Alexandria, EGY

**Keywords:** medication poisoning, chemical poisoning, children, caregivers, alexandria poison center, acute poisoning

## Abstract

Acute poisoning in children is a major public health problem worldwide. Children poisoning ranks among the top unintentional injuries in children aged less than four years. This paper aimed to describe the pattern and characteristics of acute poisoning incidents, estimate the percentage of medication poisoning among those children and highlight the possible risk factors. All children aged below 10 years admitted to Alexandria Poison Centre (APC) with acute poisoning from the July 1, 2022, to December 31, 2022, were included in the study. A pre-designed structured interviewing questionnaire was used to collect data: socio-demographic data of the poisoned child and his/her caregiver, medical history of the poisoned child and family members, history of previous poisoning incidents in the family, details of the poisoning incident including causative agent, route of poisoning, scene of poisoning, time interval to reach APC and the first aid done.

350 children admitted to APC were included in our study, of which 59% (n=208) of poisoned children were males with mean age 3.14 ± 2.28 years. The types of poisoning found were 46.6% chemical compounds, 31.4% medication, 18% household and 4% food poisoning. Most of the children were poisoned orally. High education of caregiver, urban residence and the presence of chronic disease within a family member were significantly associated with medication poisoning while low education of caregiver, drug addiction, having chronic disease among a family member and the presence of previous poisoning accident in the family were significantly associated with poisoning with chemical compounds. The study found that acute poisoning is more common among young male children in Alexandria; the chemical compounds came first as the main source of poisoning followed by the medication poisoning.

## Introduction

Acute poisoning in children is a major public health issue that has been identified as one of the top causes of accidental mortality [1]. In the USA, 41,000 incidents of non-fatal accidental poisoning were reported among children under the age of four, accounting for 2% of all injuries among this age group [2]. According to the World Health Organization (WHO), poisoning is one of the top five causes of death from unintentional injuries among children under four years of age [3].

Several risk factors have been reported to contribute to children’s poisoning, such as low caregiver awareness and improper storage of household cleaning products [4,5]. Furthermore, natural curiosity and a lack of self-control at a young age have been identified as potential risk factors [6,7]. According to mothers' opinions, the lack of supervision was the most common cause of the poisoning accident (65%), followed by caregivers’ underestimation of child behavior (29%) [8]. According to the United States Poison Centers, ingestion was the most frequently reported route of exposure (82.5%), followed by inhalation/nasal (7.2%), dermal (7%), and ocular routes (4%) [9]. 

There are many types of readily accessible substances that can be potentially toxic such as medications, topical preparations, household cleaning products, pesticides, natural gas, alcohol, nicotine, tobacco products, illicit substances, batteries, and personal care products [10].

Soave et al. indicated that medications were the most common substances implicated in poisoning (39%) among children, followed by household supplies (26%) and corrosives (16%), then plants and derivatives (8%), alcohol and substances of abuse (4%), pesticides (3%), and finally gases (2%) [11].

In the USA, the prevalence of opioid poisoning among children was 47%, where it was the most common substance contributing to death, followed by over-the-counter (OTC) pain, cold, and allergy medications (14%) [12]. Meanwhile, in Southwest China, most of the medication poisoning cases involved prescribed drugs such as anti-hypertensive drugs, anti-diabetic drugs, psychotropic drugs, anti-allergic drugs, contraceptives, and drugs for common cold [13].

The current study aimed to describe the pattern and characteristics of acute poisoning among the children admitted to the Alexandria Poison Center (APC), estimate the proportion of medication poisoning among those children, and highlight possible associated risk factors.

## Materials and methods

A cross-sectional study was conducted among children aged less than 10 years admitted to APC at the Alexandria Main University Hospital with acute poisoning. APC is the only poisoning center serving Alexandria Governorate and the surrounding rural governorates.

The study was conducted over six months, from July 1, 2022, to December 31, 2022. The sample size was calculated using Epi Info 7.2.0.1, 2016 software, based on the registered data in APC of 8,300 cases of poisoning in 2021, of which 29% were children aged <10 years, using a confidence limit of 5% and a level of confidence of 95%. The minimum required sample size was 305. The study included 350 children.

The caregivers of poisoned children were interviewed. A pre-designed structured interviewing questionnaire was used to collect data regarding the following items from the caregivers: socio-demographic data of the poisoned child and his/her caregiver, medical history of the poisoned child and family members, history of previous poisoning incidents in the family, details of the poisoning incident, including the causative agent, route of poisoning, scene of poisoning, time interval to reach APC, and first aid done [14].

Ethical considerations

The researcher sought the approval of the Ethics Committee of the High Institute of Public Health for conducting the research. They complied with the International Guidelines for Research Ethics. Verbal consent was taken from the study participants after explanation of the purpose and benefits of the research. Anonymity and confidentiality were assured and maintained. There was no conflict of interest.

Statistical analysis

The collected data were revised for accuracy and completeness, coded, and analyzed using Statistical Package for Social Sciences (SPSS) version 23 software for tabulation and analysis [15].

Qualitative data was described and analyzed in numbers and percentages using SPSS version 23 software. Quantitative data were described using range (minimum and maximum), mean, standard deviation, median, and interquartile range (IQR). The Kolmogorov-Smirnov test was used to verify the normality of the distribution.

Three regression models were created using three dependent factors: the first aid done by the caregiver, medication poisoning, and chemical poisoning. The following factors were used to create the models: socio-demographic data on the child and their caregivers. The significance of the obtained results was judged at the 5% level (p<0.05).

## Results

The cross-sectional study was carried out among 350 children diagnosed with acute poisoning and presented to the APC. More than half (59%) of the cases were male; the mean age of studied children was 3.14 ± 2.28 years. The poisoned child was the second child in the family in 33% of cases, while only 15% of the children ranked first. Nearly all children (98%) didn’t complain of any chronic diseases. Concerning the caregivers, the majority (96%) of the caregivers accompanying their children were the parents; their mean age was 27.7 ± 5.7 years old. Two-fifths of them (41%) had a secondary education level, and nearly a fifth (22%) had a university educational level. The urban residence was predominant (85%). About two-thirds (64%) had three or more children, while only 8% had one child, including the poisoned case. About one-quarter (24%) of the caregivers had chronic diseases.

Only 5% of the caregivers stated that there were previous poisoning incidents in the family, most commonly (89%) poisoning among siblings of the admitted case; meanwhile, 11% of the admitted children were previously admitted for poisoning incidents.

Description of the poisoning incident

**Figure 1:**
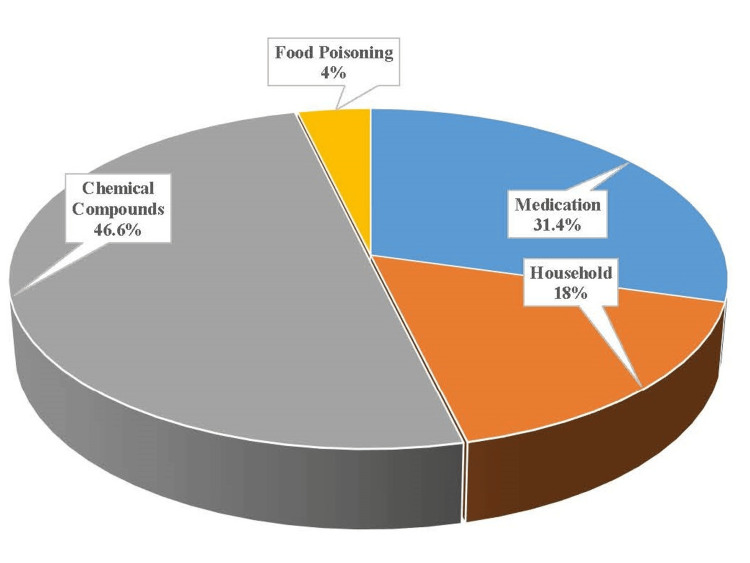
Distribution of the poisoned children attending APC according to the cause of poisoning, Alexandria, Egypt, 2023 APC: Alexandria Poison Centre

Figure 1 represents the causes of child poisoning. Less than half (47%) of the children were poisoned with chemical compounds, nearly one-third (31%) were poisoned with medications, 18% were poisoned with household cleaners, and only 4% had food poisoning.

Insecticides account for the highest proportion (38%) of poisoning with chemical compounds, while opioids, corrosive materials, and benzene each represent nearly one-fifth (22%, 21%, and 19%, respectively).

**Figure 2:**
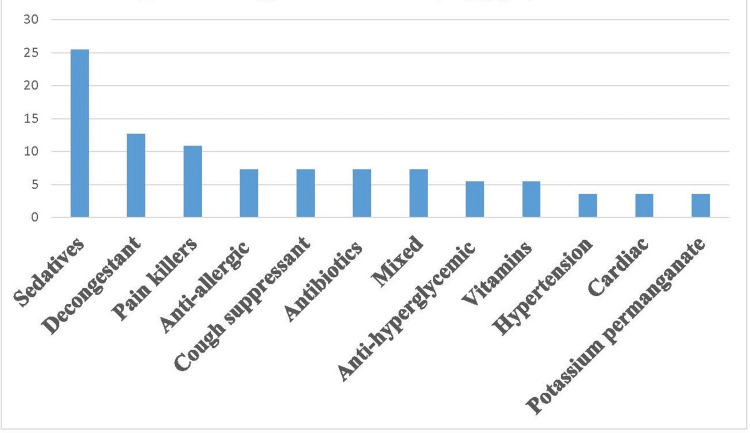
Distribution of the poisoned children attending APC according to the medications causes poisoning, Alexandria,Egypt,2023 APC: Alexandria Poison Centre

Figure 2 shows the drug categories implicated in medication poisoning. Sedatives represent about a quarter (26%) of the incidents, while poisoning with decongestants and painkillers represented 13% and 11%, respectively. 7% of the cases were poisoned by mixed categories of medications, while anti-allergic, cough suppressant, and antibiotics were almost reported equally (7%). Children poisoned with vitamins, cardiac medications, hypertensive medications, potassium permanganate, and anti-hyperglycemic medications represent less than 10% of the medication-poisoned cases. Most of the cases (91%) were poisoned orally, and only 8% were poisoned through inhalation with bronchospasm dilators.

More than half (57%) of the cases reached APC within 10-30 minutes after the poisoning incident, while 43% reached APC after 30 minutes more. As for the first aid done by the caregivers, 44% reported induction of emesis for the poisoned child, while nearly one-third (32%) gave water, 29% went directly to APC, and only 9% presented milk to their children.

Table 1 represents the factors that were associated with the children’s medication poisoning using univariate analysis while Table 2 represents the multivariate analysis. High level of education (high school, diploma, or university) of the caregiver, urban residence, and the presence of chronic disease within a family member were significantly associated with medication poisoning (odds ratio (OR) = 1.785, p = 0.041), (OR = 4.228, p = 0.01), and (OR = 2.145, p = 0.005), respectively.

The factors associated with chemical compound poisoning are presented in Table 3 using the univariate analysis and Table 4 we used the significant data to build a multivariate analysis. Low level of education (primary or middle) of caregivers, drug addiction, the presence of chronic diseases within a household family member, and the history of poisoning incidents within the same family were significantly associated with poisoning with chemical compounds (OR = 2.076, p = 0.005), (OR = 7.596, p = 0.011), (OR = 0.495, p = 0.011), and (OR = 0.127, p = 0.008), respectively.

## Discussion

The mean age of children in the current study was 3.14 ± 2.28 years, and the majority were males. This corresponds with another study in Alexandria, Egypt, in which children aged (3-5 years) and the ratio between males and females represented 1.2:1, as the males are more curious and cannot differentiate dangerous situations while exploring their surrounding environment [16]. Additionally, they have well-developed skills to locate and ingest liquids and solids but are unable to differentiate rapidly between edible liquids and solids from toxic ones [17]. 

In Ethiopia, Brazil, and Saudi Arabia, poisoning was more prevalent among male children, which might be explained by the nature of energetic and less obedient behaviors among boys, hence the increased likelihood of exposure to poisons [17-22].

The urban residence was more common for the poisoning incidents in the current study; this is similar to what was reported in Iran, which revealed that 75% of the patients were living in urban settings [17]. This may be because the majority of the admitted cases were from the urban regions of Alexandria. On the other hand, poisoning incidents were more prevalent in rural regions than in urban areas (66% and 34%, respectively) in an Ethiopian study, which might be related to low parental educational status in rural areas and certain chemicals, such as pesticides, being more prevalent in such areas [23].

In accordance with the present results, Egyptian and Malaysian studies showed that insecticides and pharmaceutical products were the most common sources of poisoning [10,24]. Meanwhile, studies in Italy, Saudi Arabia, and Turkey revealed that medication poisoning was the predominant cause of poisoning, followed by household products [12,14,25]. It is worth mentioning that during the lockdown era of Covid-19, the majority of the reported exposures were related to chemical compounds such as hand sanitizers and disinfectants [26-28].

Sedative medications were the most commonly reported class of drugs implicated in poisoning in the current study. Meanwhile, anti-hypertensives, cardiac medications, and potassium permanganate were the least likely medications to cause poisoning. This agreed with a study conducted in Iran, in which sedatives and hypnotics were often implicated in poisoning cases [29]. However, analgesics were the most regularly implicated drugs in the USA, where these drugs were the most commonly used by caregivers [30]. On the contrary, in Saudi Arabia, Sri Lanka, Brazil, and China, paracetamol and anti-pyretic were the most dominant causes of medication poisoning [20,31-33]. In South Korea, when comparing before and after Covid-19, it was found that antipyretics/nonsteroidal anti-inflammatory drugs and respiratory drugs were more common in the pre-Covid-19 group, whereas iron/vitamins, cardiovascular drugs, and hormones were more common in the post-Covid-19 group [34].

Most of the cases in this study (92%) were poisoned orally, and only 8% were poisoned through inhalation. This is in agreement with many studies in Saudi Arabia, Ethiopia, Czech Republic, and Malaysia, in which the most common route of poisoning was the oral route [14,18,32,35].

The interval between the poisoning incident and medical intervention is crucial to minimize further damage and complications, as reaching the hospital early resulted in the initiation of appropriate therapy early and, in turn, a good prognosis. In the current study, more than half (57%) of the cases reached APC within 10-30 minutes of the poisoning, while 43% reached APC after 30 minutes or more. This may be because they went first to the nearest emergency hospital and then were guided to the APC. In India, 60% were brought to the hospital during the first six hours, which resulted in positive therapeutic outcomes [36].

Urban residence was found to be significantly associated with medication poisoning in the current study, which agreed with Iran’s study, in which 75% of patients with medication poisoning lived in urban areas, as poisoned city residents are quickly or more frequently referred to a hospital [17].

In our study, a higher level of education among the caregivers was correlated with medication poisoning in the children, while a lower level of education was linked to chemical poisoning. Daifallah et al. reported that more than half of the mothers (55%) of medication-poisoning children had completed a high school education as their highest level of education [37]. In Egypt (Menoufia Governorate), it was found that mothers of poisoned children had at least a high school education in cases of medication poisoning [38]. Al-Ahdal et al. in Saudi Arabia found that chemical poisoning episodes increased significantly when the mother was less educated (P =0.017) [20].

Most of the low-educated mothers were housewives dealing with household products. Such substances are sold exposed and unbranded and can be stored in water or other beverage bottles in the kitchen, within easy reach of children. Also, poor living conditions, local beliefs, and low awareness of the hazards of chemicals are other risk factors associated with a low level of education and acute poisoning. On the other hand, a high level of education is related to medication poisoning as caregivers are mostly working, relying on multiple givers who may not coordinate closely on the timing of children’s dosages, which leads to medication poisoning due to the wrong dosage. In addition, the formulation of children’s medications that are designed to taste good but may entice children to take them when unsupervised, leading to accidental medication poisoning.

In the current study, it was found that the presence of chronic diseases within a family member was significantly associated with medication and chemical poisoning. This agreed with Feiz Disfani et al., who found a significant correlation between the presence of diseases or psychological illnesses in the family and the incidence of childhood poisoning (P<0.05) [39]. This could be explained by the fact that having a chronically ill family member increases the availability of medications at home and reduces attention given to young children. The pace of today’s lifestyle may prevent caregivers from immediately putting medicines out of reach of children after every use due to a rise in multigenerational households in which children may now have greater access to grandparents’ medications, and all the children's natural curiosity makes them mimic adults in taking medications [40].

The presence of an addicted family member could significantly increase the risk of childhood poisoning, as found in the present study. It agrees with Feiz Disfani et al., which found a positive correlation (OR, 4.54; 95% CI, 1.10-18.68; p<0.05) between chemical poisoning and the presence of an addict family member [39]. This could be due to the sedative effect of abuse substances, which leads to low awareness among the caregivers. Also, low supervision of the children leads to easy accessibility to poisons as the poisoning substances are always present at home in frequently used, unlocked drawers.

In the present study, the presence of previous poisoning incidents in the family was significantly associated with poisoning with chemical compounds. This is best explained by Feiz Disfani et al. and Mansori et al., as low supervision of the family and easy accessibility to the poisons didn’t improve after the first accident, and even the caregiver's knowledge and awareness about the consequences of poisoning didn’t change due to the absence of a routine program to educate and elevate the caregivers’ knowledge and practices about poisoning [39,41].

Limitations

In some cases, the accompanied guardian was one of the grandparents who did not have complete information about the accident of poisoning, which led to more time to contact the mother to get the needed details about the accident.

## Conclusions

Poisoning incidents are common among young children, more common among boys living in urban settings, where poisoning is mostly orally, and chemical compounds come in first as the main source of poisoning, followed by medication poisoning.

High level of education of the caregiver, urban residence, and the presence of chronic disease within a family member were significantly associated with medication poisoning, while low level of education of the caregiver, drug addiction, having chronic disease in one of the family members, and the presence of previous poisoning accidents in the family were significantly associated with poisoning with chemical compounds.

Caregivers should keep poisoning substances in locked closet away from children and make sure to have the phone number of the national poison center of their country. Ministry of Health and Population should make a routine educational programs to improve the level of knowledge about the prevention of poisoning and its first aids in all the primary health care units and the poison centers in the country.

## Appendices

**Table 1:** Logistic regression analysis of factors affecting the children with medication poisoning attending APC, Alexandria, Egypt, 2023 (n=110 children with medication poisoning versus n=240 children with any other cause of poisoning) OR: Odds ratio; ®: Reference group; CI: Confidence interval; APC: Alexandria Poison Centre *: Statistically significant at p ≤ 0.05 All variables with p<0.05 were included in the multivariate.

Socio-demographic data	Bivariate analysis	
	OR (95% CI)	p value
Child		
Female gender	1.494 (0.947 – 2.359)	0.085
Age	1.072 (0.974 – 1.180)	0.157
Order in family		
First	1.966 (1.039 – 3.723)	0.038^*^
Second	1.458 (0.880 – 2.418)	0.143
≥3^®^	1.000	–
Chronic disease	4.491 (0.810 – 24.896)	0.086
Caregiver's socio-demographic data		
High education	2.429 (1.460 – 4.040)	0.001^*^
Urban residence	6.453 (2.263 – 18.404)	<0.001^*^
Number of children		
Only child	2.256 (0.945 – 5.383)	0.067
Two children	1.247 (0.658 – 2.363)	0.498
Three children	0.921 (0.506 – 1.676)	0.787
Four or more children^®^	1.000	–
Family members has chronic disease	2.111 (1.275 – 3.496)	0.004^*^

**Table 2:** Logistic multivariate regression analysis of factors affecting the children with medication poisoning attending APC, Alexandria, Egypt, 2023 (n=110 children with medication poisoning versus n=240 children with any other cause of poisoning) OR: Odds ratio; ®: reference group; CI: Confidence interval; APC: Alexandria Poison Centre #: All variables with p<0.05 were included in the multivariate. *: Statistically significant at p ≤ 0.05

Socio-demographic data	^#^Multivariate	
	OR (95% CI)	p value
Order in family		
First	1.739 (0.899 – 3.363)	0.1
Second	1.560 (0.911 – 2.670)	0.105
≥3^®^	1	–
Caregiver's socio-demographic data		
High education	1.785 (1.025 – 3.109)	0.041^*^
Urban residence	4.228 (1.410 – 12.680)	0.010^*^
Family members has chronic disease	2.145 (1.261 – 3.651)	0.005^*^

**Table 3:** Logistic regression analysis of factors affecting children with chemical poisoning attending APC, Alexandria, Egypt, 2023 (children with chemical poisoning (n=163) versus children with any other cause of poisoning (n= 187)) OR: Odds ratio; ®: reference group; CI: Confidence interval; APC: Alexandria Poison Centre All variables with p<0.05 were included in the multivariate. *: Statistically significant at p ≤ 0.05

Socio-demographic data	Bivariate analysis		
	OR (95%C.I)	p	
Child's socio-demographic data			
Male gender	1.708 (1.107 – 2.636)	0.015^*^	
Age	0.982 (0.895 – 1.077)	0.693	
Order in family			
First^®^	1.000		
Second	1.165 (0.598 – 2.269)	0.654	
≥3	1.823 (0.973 – 3.413)	0.061	
Chronic disease	0.568 (0.103 – 3.144)	0.517	
Caregiver's socio-demographic data			
Low education	1.996 (1.285 – 3.100)	0.002^*^	
Rural residence	2.636 (1.410 – 4.927)	0.002^*^	
Number of children (> 2 child)	1.604 (1.027 – 2.506)	0.038^*^	
Drug addict	6.046 (1.305 – 28.010)	0.021^*^	
Chronic disease	0.528 (0.318 – 0.874)	0.013^*^	
Is there any previous poisoning accidents in the family?	0.133 (0.030 – 0.587)	0.008^*^	

**Table 4:** Logistic multivariate regression analysis of factors affecting children with chemical poisoning attending APC, Alexandria, Egypt, 2023 (children with chemical poisoning (n=163) versus children with any other cause of poisoning (n= 187)) OR: Odds ratio; ®: Reference group; CI: Confidence interval; APC: Alexandria Poison Centre #: All variables with p<0.05 were included in the multivariate. *: Statistically significant at p ≤ 0.05

Child's socio-demographic data	^#^Multivariate	
	OR (95% CI)	p
Male gender	1.512 (0.955 – 2.394)	0.078
Caregiver's socio-demographic data		
Low education	2.076 (1.247 – 3.454)	0.005^*^
Rural residence	1.551 (0.770 – 3.121)	0.219
Drug addict	7.596 (1.595 – 36.175)	0.011^*^
Chronic disease	0.495 (0.288 – 0.848)	0.011^*^
Were there any previous poisoning accidents in the family?	0.127 (0.028 – 0.583)	0.008^*^
